# Limited available evidence supports theoretical predictions of reduced vaccine efficacy at higher exposure dose

**DOI:** 10.1038/s41598-019-39698-x

**Published:** 2019-03-01

**Authors:** Kate E. Langwig, M. Gabriela M. Gomes, Mercedes D. Clark, Molly Kwitny, Steffany Yamada, Andrew R. Wargo, Marc Lipsitch

**Affiliations:** 1 Department of Biological Sciences, Virginia Tech, Blacksburg, VA 24061 USA; 2 Center for Communicable Disease Dynamics, Department of Epidemiology, Harvard T.H. Chan School of Public Health, Boston, MA 02115 USA; 3 CIBIO-InBIO, Centro de Investigação em Biodiversidade e Recursos Genéticos, Universidade do Porto, Vairão, Portugal; 4 Liverpool School of Tropical Medicine, Liverpool, L3 5QA UK; 5 Virginia Institute of Marine Science, College of William and Mary, Gloucester Point, VA 23062 USA

## Abstract

Understanding the causes of vaccine failure is important for predicting disease dynamics in vaccinated populations and planning disease interventions. Pathogen exposure dose and heterogeneity in host susceptibility have both been implicated as important factors that may reduce overall vaccine efficacy and cause vaccine failure. Here, we explore the effect of pathogen dose and heterogeneity in host susceptibility in reducing efficacy of vaccines. Using simulation-based methods, we find that increases in pathogen exposure dose decrease vaccine efficacy, but this effect is modified by heterogeneity in host susceptibility. In populations where the mode of vaccine action is highly polarized, vaccine efficacy decreases more slowly with exposure dose than in populations with less variable protection. We compared these theoretical results to empirical estimates from a systematic literature review of vaccines tested over multiple exposure doses. We found that few studies (nine of 5,389) tested vaccine protection against infection over multiple pathogen challenge doses, with seven studies demonstrating a decrease in vaccine efficacy with increasing exposure dose. Our research demonstrates that pathogen dose has potential to be an important determinant of vaccine failure, although the limited empirical data highlight a need for additional studies to test theoretical predictions on the plausibility of reduced host susceptibility and high pathogen dose as mechanisms responsible for reduced vaccine efficacy in high transmission settings.

## Introduction

Measurements of vaccine efficacy frequently vary across transmission settings, which limits generalizability of results from preclinical experiments and clinical trials. For example, efficacy of the RTS/S malaria vaccine varies dramatically among location (e.g. 17% efficacy in Burkina Faso to 66% in Kilifi, Kenya)^1^. Differences in exposure risk among individuals likely contribute to observed differences in vaccine efficacy among clinical trials^2^. Differences in exposure dose affect disease risk, and high doses of pathogens increase the probability of infection in most host-pathogen systems^3^. However, the relationship between pathogen dose and vaccine efficacy is less clear, and few studies have tested vaccines across a broad range of pathogen doses^4–10^, notwithstanding the belief that vaccines may be overcome at high rates of exposure^11^.

Differences in susceptibility among and within populations also affect risk, thus influencing measurements of vaccine efficacy. Here, we define ‘susceptibility’ as the probability of infection per unit of exposure. Myriad factors may affect susceptibility including age, nutritional status, or infection with other parasites^12^. Effective vaccines should reduce mean susceptibility thereby decreasing the probability hosts become infected. However, in addition to reducing mean susceptibility, vaccines may also change variance in susceptibility, dependent on the mode of vaccine action, which could further influence population measures of vaccine efficacy.

The mode of vaccine action, or the shape and variance of susceptibility, can influence disease dynamics, and is therefore important to consider in assessing spread and transmission of pathogens through vaccinated populations^10,13^. Vaccines that have highly polarized modes of action have been termed “all-or-nothing” because hosts are practically either completely protected or unprotected following vaccination. Modes of vaccine action that are less polarized are termed “leaky”, and in the most extreme instance, partially protect each host to an identical degree. In randomized vaccine trials, the distribution of pathogen exposure dose should be balanced by randomization between the vaccinated and control groups. However, the distribution (mean, variance and higher moments) may differ between these groups, while within a group there will be varying levels of pathogen exposure dose^10^. Here, we examine the impact of variance in susceptibility in vaccinated and unvaccinated individuals on vaccine efficacy across escalating pathogen doses.

## Methods

We examined the influence of exposure dose and heterogeneity in susceptibility on vaccine efficacy. We first sampled susceptibility of a host population from a beta distribution with a mean susceptibility of 0.18 for the vaccinated group, and 0.9 for the unvaccinated (control) group, arbitrarily assuming a total number enrolled in the trial of 140,000 (70,000 in each arm). Mean susceptibility values were selected to obtain 80% efficacy at the limit of a low exposure-dose, with a visible decline over the range of doses considered. We then selected a group of 10,000 individuals from both the vaccinated and control groups to challenge at a given dose. From this, we calculated the probability of each individual becoming infected at a given dose using 1 – exp(-dose*susceptibility), where both dose and susceptibility were continuous variables. We performed this simulation for nine different susceptibility shape combinations, with variance ranging from 0.09 (polarized beta distribution) to 9 × 10^−7^ (homogenous distribution where nearly every individual had a susceptibility equal to the mean). We then took the mean probability of infection for each dose and estimated vaccine efficacy at each using 1 – Risk Ratio (the probability of infection in the vaccine group divided by the probability of infection in control group). Under this model, the probability of becoming infected depends only on susceptibility and the total exposure dose, and therefore exposure could be considered as either dose during a single exposure, or the total number of exposures of fixed or varying dosage.

We compared our results with published estimates of vaccine efficacy obtained from controlled challenge studies based on a systematic literature review using keywords (dose AND vaccine efficacy) OR (dose AND vaccine probability) in PubMed (Fig. S1). Two observers screened the PubMed results, and we restricted our comparisons to studies where vaccinees (vaccinated individuals) and an unvaccinated control group were tested against multiple pathogen doses in the same study, and sample sizes were greater than 2 individuals per group. We also identified several additional studies meeting our criteria based on reviews of reference sections of identified articles. Several studies we identified also varied vaccine dosage, and in cases where vaccine dose did not significantly influence vaccine protection, we combined data among treatment groups. In all other instances, we calculated vaccine efficacy of the most efficacious vaccine type at the most efficacious vaccine dose reported in each study. We analyzed the effect of pathogen dose on vaccine efficacy using a generalized linear mixed model with a binomial distribution and a logit link with disease as a random effect and treatment group interacting with ordinal dose as fixed effects. Statistical models were analyzed using package lme4^14^, and all simulations and analyses were conducted in RStudio v. 1.0.153^15^.

## Results

**Figure 1:**
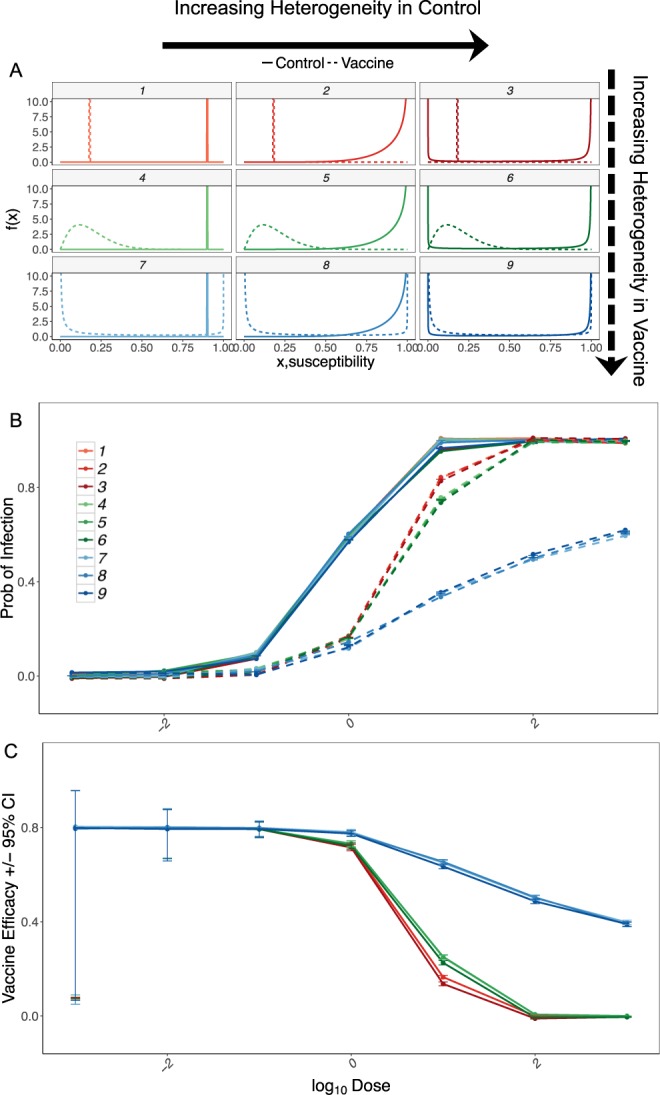
Estimates of vaccine efficacy over a range of pathogen doses allowing for variation in heterogeneity in susceptibility. (**A**) Susceptibility distributions of vaccinated and control (unvaccinated) groups used in simulation. (**B**) Estimated probability of infection control (solid lines) and vaccine group (dashed lines) +/− standard error of mean. Colors correspond to susceptibility distributions in panel (**A**). (**C**) Measurable vaccine efficacy decreases with increasing exposure dose despite the constancy in intrinsic efficacy given by the susceptibility distributions (intrinsic vaccine efficacy = 0.8). Colors and labels are the vaccine efficacy estimated from the corresponding susceptibility distributions in panel (**A**). Error bars show 95% confidence intervals.

Simulations showed that vaccine efficacy decreased non-linearly with increasing dose (Fig. 1). As dose increased, vaccine efficacy declined most sharply when the susceptibility distributions in the vaccinated group had lower variance. At moderate doses, highly polarized susceptibility among vaccinees resulted in the highest estimates of vaccine efficacy. At low exposure doses, there was little difference in probability of infection and vaccine efficacy if susceptibility was homogeneous or hump-shaped, despite a three orders of magnitude difference in variance. At the highest doses, vaccine efficacy was close to 0 for groups with homogeneous or hump-shaped modes of vaccine action, whereas vaccine efficacy remained above 40% for polarized distributions. Heterogeneity in susceptibility in the control group had little influence on probability of infection and vaccine efficacy because the mean probability of infection was very high, and no individuals were entirely protected.

**Figure 2:**
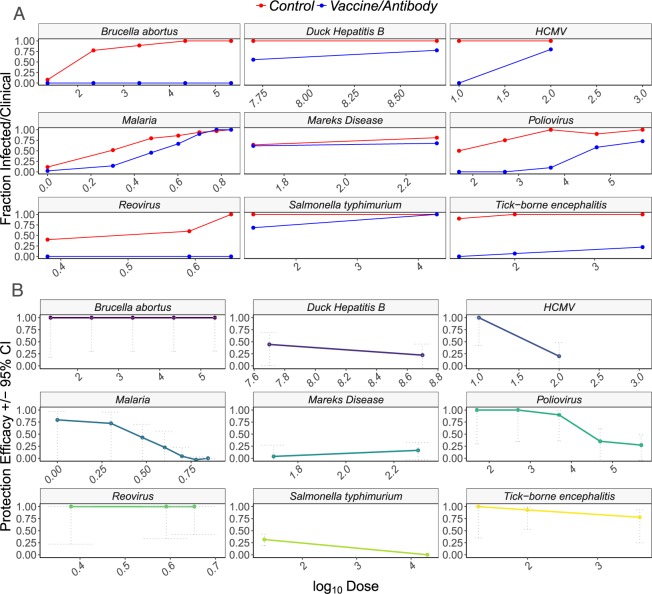
The fraction of individuals infected (**A**) and estimates of vaccine efficacy (**B**) for *Brucella abortus* in mice (H.38 *B. melitensis* killed vaccine^25^), duck hepatitis b virus in ducks (duck hepatitis b virus surface protein DNA vaccine^26^), Human cytomegalovirus in humans (Towne cytomegalovirus vaccine^27^), malaria in mice (anti-circumsporozoite protein, efficacy estimated from total residual sporozoite scores across all bites binned across groups for visualization^28^), Marek’s disease in broiler chickens (turkey herpesvirus vaccine^5^), poliovirus in human infants (oral poliovirus vaccine^29^), reovirus in chickens (attenuated reovirus vaccine^30^), *Salmonella typhimurium* in mice (heat-killed *S. typhimurium* vaccine^7^), and tick-borne encephalitis in mice (tick-borne encephalitis vaccine^31^).

Our systematic review highlighted the paucity of studies that have tested vaccines at multiple pathogen doses. Our broad search criteria identified 5389 articles (as of March 23 2017). Of these, 253 articles were identified for further screening based on information in the abstract, however only 16 studies met the inclusion criteria (Table 1). Nine studies used infection or clinical signs as an outcome variable whereas the others used mortality (Fig. 2). Nine total studies also tested vaccines across more than two different pathogen doses, but only five of these used infection or clinical signs as a response variable.

**Table 1:** Studies identified as a result of a systematic review of vaccines tested over multiple pathogen challenge doses. Inclusion criteria included studies where vaccinees (vaccinated individuals) and an unvaccinated control group were tested against multiple pathogen doses in the same study. *Indicates a study in which only two pathogen doses were tested for a seronegative participant group, but three doses were tested across vaccinees.

Study	No. of Challenge Doses	Study Organism	Pathogen	Response Variable	Citation
Bosseray 1980	5	Mouse	*Brucella abortus*	Placental colonization	^25^
Bublot 2007	6	Chicken	H5N1 Influenza	Mortality	^32^
Chernokhaeva 2016	3	Mouse	Tick-borne encephalitis virus	Infection and Mortality	^31^
Churcher 2017	4	Mouse	Malaria	Infection	^28^
Cronly-Dillon 1972	2	Mouse	*Salmonella* sv. *typhimurium*	Infection	^7^
Delagrave 2012	4	Mouse	Herpes simplex virus Type 2	Mortality	^33^
Ghiasi 1997	2	Mouse	Herpes simplex virus Type 1	Mortality	^34^
Henry 1966	5	Human	Poliovirus	Infection	^29^
Hatch 1964	12	Mouse	*Francisella* (*Pasteurella*) *tularensis*	Mortality	^35^
Islam 2007	2	Chicken	Mareks Disease virus	Infection	^5^
Marchart 2003	3	Mouse	*Pasteurella multocida*	Mortality	^8^
Miller 2006	2	Duck	Duck hepatitus B virus	Chronic infection	^26^
Plotkin 1989	2*	Human	Human cytomegalovirus	Infection	^27^
Sebunya 1982	3	Mouse	*Haemophilus pleuropneumoniae*	Mortality	^36^
van Loon 2002	3	Chicken	Reovirus	Isolation from organ	^30^
Yamashita 2009	2	Sevenband grouper	Red-spotted grouper nervous necrosis virus	Mortality	^37^

Empirical estimates showed that increasing pathogen challenge dose decreased infection vaccine efficacy (treatment and dose interaction: P < 0.0001), although results varied among studies (random effect of disease: variance = 3.43). For some diseases that examined vaccine protection across five or more different pathogen challenge doses (e.g. poliovirus and malaria), vaccine efficacy decreased in accordance with our predictions from the simulation. However, for *Brucella abortus* and reovirus, which used organ colonization as the response variable, there was no decrease in vaccine efficacy with increasing pathogen dose. While the overall trend aligned with our theoretical predictions, most studies tested a small number of pathogen doses making it difficult to discern whether vaccines were truly not overcome at high pathogen challenge doses or whether the doses studied were just insufficient to overcome vaccines.

## Discussion

Our simulation suggests that estimates of vaccine efficacy should be much lower under high pathogen challenge dose regardless of susceptibility heterogeneity. While our review revealed a very small number of studies that have tested vaccines across a broad range of pathogen challenge doses, there is some limited empirical evidence that data aligns with our theoretical predictions. However, two studies appeared to show no consistent trend with dose, and offered hosts complete protection regardless of pathogen dose. Notably, doses of *Brucella abortus* spanned five orders of magnitude, but no vaccinated hosts were colonized across this large dose range. These findings suggest that these vaccines drastically reduce susceptibility above the range tested in our simulation and highlight a need for additional experiments testing highly efficacious vaccines over multiple pathogen doses.

Additional dose-ranging challenge studies would be useful in that they can be used to both assess vaccine efficacy^2,16^ and estimate heterogeneity in host susceptibility^10,13^. These estimates of host heterogeneity can be incorporated into dynamical models to improve predictions of pathogen spread in vaccinated populations^10^, and also account for the apparent reductions in vaccine efficacy over time in clinical trials^2^. While human-challenge studies are ethically controversial and only appropriate for some pathogens, animal systems can still provide important insights about the failure of vaccines to protect individuals in high transmission settings, and our review suggests that there is opportunity to rapidly expand theory given the limited data on this topic^17^. While extrapolation to human systems should be done with care, these studies would help to increase the fundamental understanding of the relationship between host susceptibility, pathogen dose, and vaccine failure.

Across pathogen species and strains, previous studies have noted that pathogens with higher virulence have lower infective doses, suggesting the potential for interactions between low-dose vaccine efficacy and pathogen virulence^18,19^. While our study examined vaccine efficacy within pathogen species and strains, future work could examine similar relationships across pathogen strains to assess whether vaccine protection is higher at low doses regardless of strain virulence. If vaccines block low dose challenges in both virulent and avirulent strains, this may influence strain competition and pathogen evolution^20^. However, experiments including clinical trials involving infections that are not always symptomatic (thus in which some infections may go undetected) can produce biased results, and it will be important to design such studies carefully to avoid conflating differences in efficacy with differences in the proportion of infections that are detected^21^.

Although our simulation showed that increasing pathogen challenge dose decreased vaccine efficacy, variance in susceptibility also affected vaccine protection. When the mode of vaccine action was polarized (e.g. approaching “all-or-nothing”), vaccine protection decayed more slowly with increasing dose. Similar results are expected if exposure accumulates over time and 1 – Rate Ratio is used as the measure of vaccine efficacy^16^. Interestingly, vaccine protection was similar when distributions of susceptibility were both homogeneous and hump-shaped. The larger change in vaccine protection when susceptibility distributions were highly polarized is likely a consequence of the inclusion of numerous individuals of very low (near 0) susceptibility. Nonetheless, the mode of vaccine action is frequently poorly understood but often assumed to be bimodal, and our results underscore the importance of heterogeneity in susceptibility in influencing vaccine efficacy, particularly in high exposure settings. Understanding the distribution of vaccine protection, rather than just the mean level, can help to improve predictions of vaccine efficacy in heterogeneous populations^10^.

Studies often speculate about the potential for vaccine failure at high pathogen doses^11,22^, yet prior to this study, there appears to be little empirical or theoretical basis for this claim. Our results highlight a theoretical basis and some empirical evidence for reductions in vaccine efficacy at high exposure doses, and also reveal the strong potential for heterogeneity in susceptibility to influence vaccine efficacy. Differences in host susceptibility, as have been noted in recent studies on influenza^23^ and mumps^24^, can explain age-stratified infection dynamics or disease resurgence that is often attributed to vaccine failure. As both pathogen dose and host susceptibility likely play important roles in disease dynamics in vaccinated populations, disentangling these effects should be an important priority in predicting the public health impact of vaccination programs.

## Supplementary information

Figure S1

## Data Availability

All data files are referenced in the manuscript in Table 1 and Fig. 2. Data within those files is available on https://github.com/klangwig/vaccineANDpathogen_dose.
