# Supplementary material for: Limited available evidence supports theoretical predictions of reduced vaccine efficacy at higher exposure dose
Source: Sci Rep. 2019 Mar 1;9:3203. doi: 10.1038/s41598-019-39698-x (PMC6397254; doi:10.1038/s41598-019-39698-x)
Supplement: Supplementary file 1 — Figure S1 [file 41598_2019_39698_MOESM1_ESM.docx]

Supplemental material

***Limited available evidence supports theoretical predictions of reduced vaccine efficacy at higher exposure dose***

Kate E. Langwig, M. Gabriela M. Gomes, Mercedes D. Clark, Molly Kwitny, Steffany Yamada, Andrew R. Wargo, Marc Lipsitch

Figure S1. The Preferred Reporting Items for Systematic Reviews and Meta-Analyses (PRISMA) Flow Diagram.

Studies included in quantitative synthesis
(n = 9)

Records retained from title and abstract
(n = 253)

Full-text articles excluded, with reasons
(n = 71)

1. Only a single pathogen dose was tested (n = 36)

2. Multiple pathogen doses used, but experimental conditions were different (n = 19)

3. Study indicated multiple doses were used, but doses were not reported (n = 5)

4. A dose-ranging challenge occurred in a single group (n = 6)

5. No control group (n=2)

6. Fewer than 3 individuals per group (n=3)

Additional records identified through other sources
(n = 8)

Studies included in qualitative synthesis
(n = 16 )

Full-text articles assessed for eligibility
(n = 87 )

Records excluded (n = 5136)

Records after duplicates removed
(n = 5389)

## Identification

## Eligibility

## Included

## Screening

Records identified through database searching: PubMed
(n = 5381)
